# New Insights into the Genomic Organization and Splicing of the *Doublesex* Gene, a Terminal Regulator of Sexual Differentiation in the Silkworm *Bombyx mori*

**DOI:** 10.1371/journal.pone.0079703

**Published:** 2013-11-07

**Authors:** Jianping Duan, Hanfu Xu, Huizhen Guo, David A. O'Brochta, Feng Wang, Sanyuan Ma, Liying Zhang, Xingfu Zha, Ping Zhao, Qingyou Xia

**Affiliations:** 1 State Key Laboratory of Silkworm Genome Biology (Southwest University), Chongqing, PR China; 2 Henan Provincial Key Laboratory of Funiu Mountain Insect Biology, Nanyang Normal University, Nanyang, PR China; 3 Department of Entomology, University of Maryland, College Park, United States of America; International Centre for Genetic Engineering and Biotechnology, Italy

## Abstract

Sex-determination mechanisms differ among organisms. The primary mechanism is diverse, whereas the terminal regulator is relatively-conserved. We analyzed the transcripts of the *Bombyx mori* doublesex gene (*Bmdsx*), and reported novel results concerning the genomic organization and expression of *Bmdsx*. *Bmdsx* consists of nine exons and eight introns, of which two exons are novel and have not been reported previously. *Bmdsx* transcripts are spliced to generate seventeen alternatively-spliced forms and eleven putative trans-spliced variants. Thirteen of the alternatively-spliced forms and five of the putative trans-spliced forms are reported here for the first time. Sequence analysis predicts that ten female-specific, six male-specific splice forms and one splice form found in males and females will result in four female-specific, two male-specific Dsx proteins and one Dsx protein common to males and females. The Dsx proteins are expected to be functional and regulate downstream target genes. Some of the predicted Dsx proteins are described here for the first time. Therefore the expression of the *dsx* gene in *B. mori* results in a variety of *cis*- and trans-spliced transcripts and multiple Dsx proteins. These findings show that in *B. mori* there is a complicated pattern of *dsx* splicing, and that the regulation of splicing and sex-specific functions of lepidopteran *dsx* have evolved complexity.

## Introduction

In insects sex is determined autonomously in somatic cells and the mechanisms that govern somatic sexual differentiation have been studied in many species. What has emerged from these studies is the realization that there are a diverse array of primary upstream signals in these various sex-determination hierarchies whereas the downstream genes in these hierarchies are relatively few and comparatively well-conserved [1]. The large number of diverse insects for which we have a molecular genetic understanding of the sex-determination hierarchy confronts us with the challenge of understanding how much divergence of the participating genes can be tolerated while maintaining functional conservation.

Sex-determination hierarchies have evolved in a retrograde manner from bottom to up as first proposed by Wilkins (1995). There are myriad primary mechanisms initiating the sex-determination cascade, such as the X:A ratio in *D. melanogaster* [2], the dominant maleness factor on Y chromosome in *C. capitata* [3], the haploidy/diploidy and the number and allelic forms of the *csd* gene in *A. mellifera* [4], the activity of the *zsd* gene in *N. vitripennis* [5], and the dominant epistatic factor on W chromosome in *B. mori* [6,7] and so on. Unlike the diversity of genes involved in the upper part of the sex determination cascade, the genes and functions involved in the lower parts of the cascade are relatively well-conserved [8]. For example, tra (transformer)-mediated expression of *dsx* (*doublesex*) is prevalent in most dipterans and well-studied hymenopterans, however only in drosophilids is *Sxl* (*Sex lethal*) the master regulator of the *tra/dsx* mode of sex determination [9-11]. In these insects *Sxl* acts as the master gene responsible for initiating and maintaining the choice of sexual identity via an autoregulatory feedback loop [1,9-14]. In several dipterans and hymenopterans such as *C. capitata*, *A. mellifera* and *N. vitripennis Sxl*’s place upstream of *dsx* is occupied by *tra* homologues, so that *tra* initiates the choice of sexual identity and maintains that choice through autoregulation [5,10,15,16]. Both *Sxl* and *tra* genes are evolving rapidly, with *tra* being relatively more conserved than *Sxl*. So far, the homolog of *tra* in *B. mori* has not been identified, and whether the *B. mori* homolog of *Sxl* serves a sex-determining role remains unclear [17]. *Sxl* in *D. melanogaster* produces three early transcripts and seven late, sex-specific transcripts encoding multiple related polypeptides [18] whose functions are still unclear. *Sxl* in *B. mori* is not sex-specifically expressed, as is the case in other non-Drosophilid species [10,11], and therefore its role may also be similar in these species, although it appears not to act as the master regulator.

The *dsx* gene is the terminal regulator of sex differentiation at the bottom of the sex-determination cascade and its structure, expression and function are conserved among organisms [19]. The *Drosophila dsx* gene controls the development of sexually dimorphic features by producing two alternatively-spliced transcripts encoding two-related sex-specific Dsx proteins with common amino termini but sex-specific carboxy termini [20-24]. In addition, two transcripts are present in both sexes throughout the larval period [25]. This is the case in *M. domestica* and *A. mellifera* in which in addition to sex-specific transcripts there is a *dsx* transcript encoding a common Dsx protein in males and females [26,27]. In *Aedes aegypti*, *A. assama*, *A. mylitta* and *B. mori* a second female-specific Dsx protein is predicted [28-30]. Recently, we proposed that *B. mori* has a third female-specific Dsx protein predicted to arise from a novel trans-spliced transcript, *Bmdsx-dsr2*d [31]. The extent to which insects have evolved multiple sex-specific Dsx proteins warrants further study.

To date relatively few *dsx* splice forms and proteins have been described. The only exception to date has been in *A. assama* in which the *dsx* homologue results in six alternatively-spliced forms in females, resulting in two female-specific Dsx proteins [29]. Additionally, why ectopic expression of female Dsx proteins (DsxF) in male *M. domestica* [26] and *B. mori* [31,32] did not show any signs of morphological sex reversal has not been adequately explained. One possible reason might be a requirement for a female-specific cofactor for DsxF to promote female development and the existence of an antagonistic effect of the male form of Dsx, DsxM. So, while the general framework of *dsx* expression and Dsx function are fairly well established there are aspects that remain unclear. Here we present a comprehensive analysis of *Bmdsx* expression in conjunction with an analysis of the resulting transcripts that show a complex pattern of expression and splicing that has not been described before. The biological implications of this complex pattern of *Bmdsx* splicing remain to be determined, however it is certain to enlighten efforts to understand insect sex determination and its evolution. 

## Materials and Methods

### PCR with exon-specific primers

Total RNA was extracted from the head, integument, hemocytes, midgut, fat body, trachea, malpighian tubules, silk glands and gonads of both sexes using Trizol reagent (Invitrogen, USA) according to the manufacturer’s protocol. cDNA templates were synthesized by reverse transcription using M-MLV reverse transcriptase (Promega, USA) as described [31]. PCR amplifications were performed using primer set P1/P2 to assess the expression of *Bmdsx* in day-3 fifth-instar larvae of both sexes. The relative abundance of each band was quantified by densitometric measurement of the EB-stained gel using Quantity One® software (Bio-Rad, USA). P1 and P2 bound specifically to exons 1 and 6, respectively which are common to *Bmdsx* transcripts in both males and females. The following conditions were used: 94°C, 3 min followed by 30 cycles of 94°C, 30 s; 57°C, 40 s; and 72°C, 1 min; with a final 10 min extension at 72°C. The *B. mori* cytoplasmic actin 3 gene (*Bmactin3*) was used as the reference gene.

### 3' RACE, sequencing validation and protein analysis

3' RACE was conducted as described [31] for two successive rounds of PCR amplification, initially with P3/3P1 and then with P4/3P2 primer pairs. 3' RACE was performed using GeneRacer^TM^ according to the manufacture’s recommendations (Invitrogen, USA). GeneRacer^TM^ Oligo-d(T) was used to initiate the reverse transcription reaction while primers P3 and P4 were specific to the 5'-terminal region of *dsx* transcripts. All amplified products were cloned and sequenced using routine methods. Sequencing data have been deposited in the GenBank under the accession numbers KF255805-KF255831. These sequencing results were then compared by BLAST analysis against the BGI *B. mori* genome assembly (http://silkworm.swu.edu.cn/silkdb/) [33,34] to confirm novel transcripts and genomic organization of *Bmdsx*. Transcripts were virtually translated and the predicted proteins were aligned using ClustalX [35] and GeneDoc [36] for analysis.

### RT-PCR analysis

RT-PCR reactions were performed using cDNA templates synthesized as described above and the position-specific primers listed in Table 1. Primers P1, P3 and P4 were specific to exon 1. Primers P5, P9 and P2 were specific to exons 3n, 6n, and 6, respectively. Primers P6 and P8 were specific to the 15-bp and 133-bp stretches in exons 3 (exon 3s) and 4 (exon 4s), respectively. Primer P7 was specific to exon 3/4, a splicing product between the 3' end of unstretched exon 3 and the 5' end of unstretched exon 4. Primers P1 was combined with primers P5, P6, P7, P8 and P9 to detect the alternative-splicing of exons 3n, 3s, 3/4, 4s and 6n, respectively. The PCR conditions were used as described above, using some modifications of the annealing temperatures according to Table 1.

**Table 1:** Primers used for PCR and 3'RACE.

Primer	Sequence	Annealing temperature (℃)
P1	TCCTCGTGCCCCTCCTAACT	58
P2	GAAGTTGACGGGGAATCGGT	56
P3	TGTCACCATACGCTCCACCG	58
P4	CCACTGATACCACCCCCGCA	60
3P1	GCTGTCAACGATACGCTACGTAACG	60
3P2	CGCTACGTAACGGCATGACAGTG	60
P5	TGAAAGGAAAGACCCAAACC	52
P6	CCAGCATTTTCTATTAAAGTCC	50
P7	TTTCCAGCATTTTCTGGCG	52
P8	TCACACACTGGCACATCACT	55
P9	CTCCATTGACATAATCCAGG	50

## Results and Discussion

### Unexpected alternatively-spliced and trans-spliced *Bmdsx* transcripts

**Figure 1:**
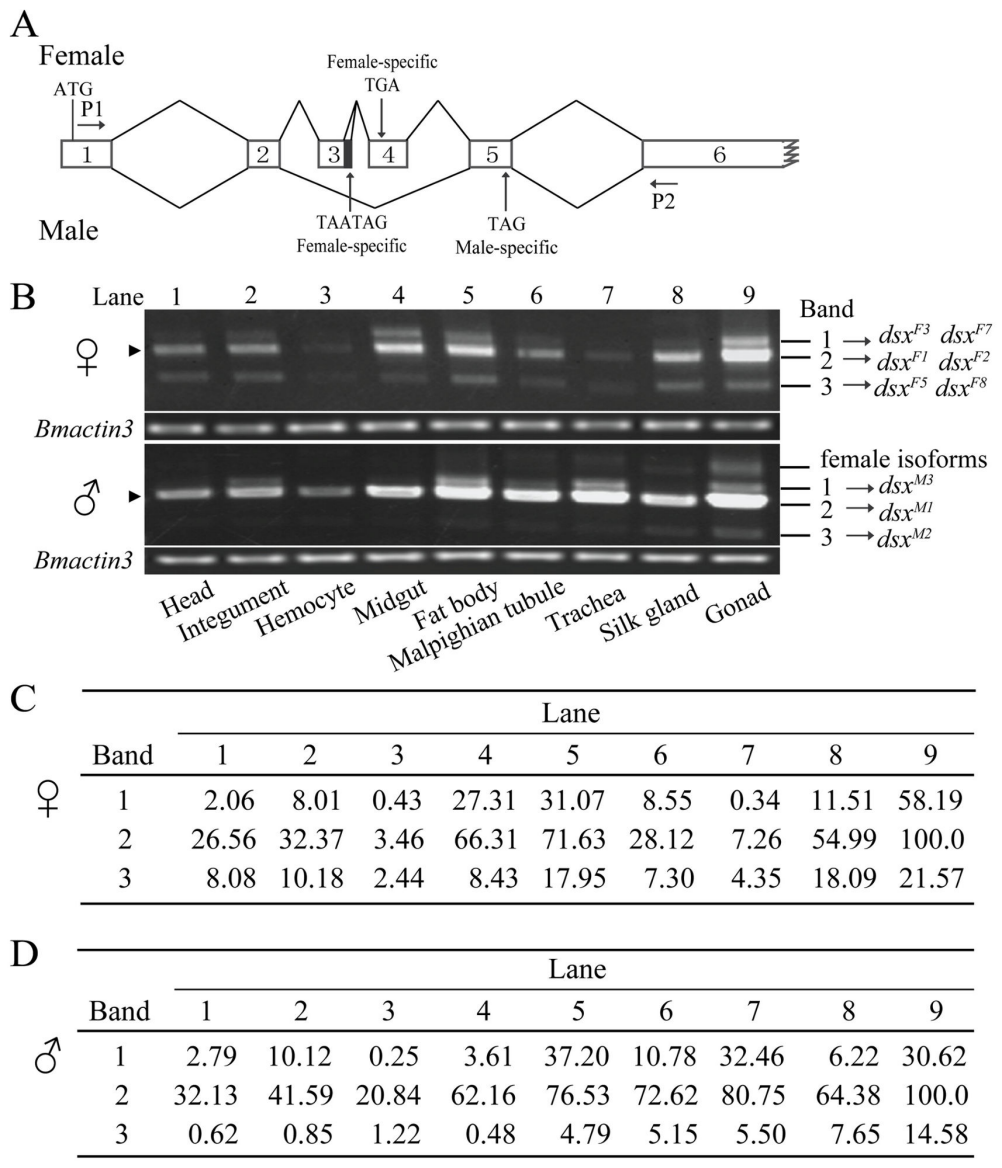
Unexpected expression of *Bmdsx* in tissues from males and females. (A) Previously-reported structure of *Bmdsx*. Horizontal arrow indicates the position of the primers used for expression analysis of *Bmdsx*. Vertical arrows show the positions of stop codons. The black box is the stretch of 15 bp in exon 3. Exons 1, 2, 5 and 6 are common to both sexes, and exons 3 and 4 are female-specific. (B) Expression analysis of *Bmdsx* in tissues from males and females. Arrowheads shows the positions of the splice forms previously-reported. *Bmactin3* was used as the internal control for message abundance. (C) The relative abundance (%) of each amplification band corresponding to the tissues of females. (D) The relative abundance (%) of each amplification band corresponding to the tissues of males.

**Figure 2:**
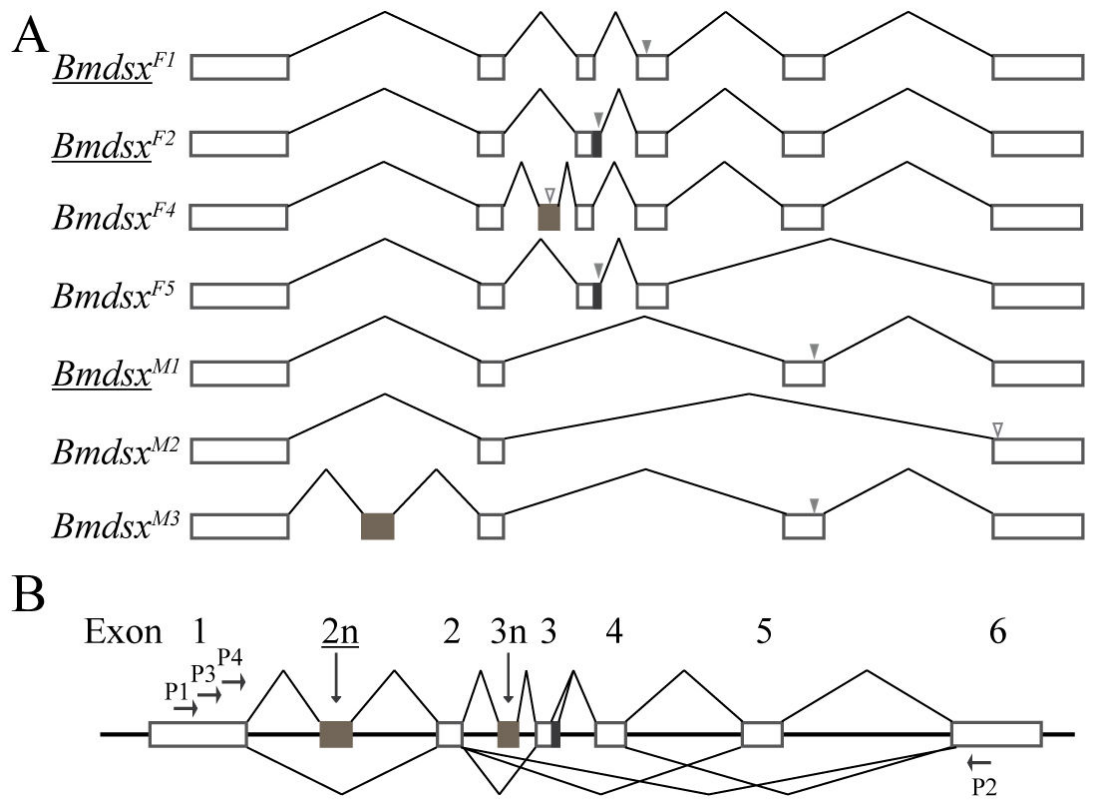
Novel splice forms and exons of *Bmdsx*. (A) Splice forms of *Bmdsx* discovered while sequencing PCR-amplification products recovered during the expression analysis in Figure 1B. Underlined splice forms were previously-reported sex-specific splice forms. Open boxes represent *Bmdsx* exons, and the “V” lines indicate introns. Black and brown boxes are a stretch of 15 bp in exon 3 and novel exons, respectively. Grey triangle shows the reported stop codon, and hollow triangle indicates the newly-gained stop codon of the novel splice form expected to be degraded by nonsense-mediated degradation (NMD). (B) First revised gene model and expression pattern of *Bmdsx*. Vertical arrows point to novel exons 2n and 3n upstream of the exons 2 and 3, respectively. Underlined exon number refers to the novel exon reported previously as part of the trans-spliced variant *Bmdsx-dsr2*d. P1/P2 is the primer set used for expression analysis. P3 and P4 are the primers used for 3'RACE.

Earlier studies reported that *Bmdsx* transcripts are spliced in a sex-specific manner to yield two female forms (*Bmdsx* ^*F1*^and *Bmdsx* ^*F2*^) and one male form (*Bmdsx* ^*M*^) [19,30]. In a recent study, we predicted the existence of a novel exon, 2n, and a third female-specific splice form *Bmdsx* ^*F3*^ [31]. To confirm this mode of splicing and expression, PCR was performed using the primer set P1/P2 binding specifically to the exons 1 and 6 that are common to all splice variants (Figure 1A). An unexpected expression pattern was observed showing extra amplification bands in both sexes, in addition to the bands predicted based on earlier studies (Figure 1B), indicating that some unidentified splice forms of *Bmdsx* remained to be described. Subsequently, all the comparatively high-abundance bands were cloned and sequenced. This analysis identified four female-specific (*Bmdsx* ^*F1*^, *Bmdsx* ^*F2*^, *Bmdsx* ^*F4*^ and *Bmdsx* ^*F5*^) and three male-specific (*Bmdsx* ^*M1*^, *Bmdsx* ^*M2*^ and *Bmdsx* ^*M3*^) splice forms (Figure 2A). *Bmdsx* ^*F1*^, *Bmdsx* ^*F2*^and *Bmdsx* ^M1^ are well-known alternatively-spliced forms described in earlier studies, while transcripts *Bmdsx* ^*F4*^, *Bmdsx* ^*F5*^, *Bmdsx* ^*M2*^ and *Bmdsx* ^*M3*^ have not been described previously. *Bmdsx* ^*F5*^ and *Bmdsx* ^*M2*^ are identical to *Bmdsx* ^*F2*^ and *Bmdsx* ^*M1*^, respectively, except for the absence of exon 5. *Bmdsx* ^*F4*^ differs from *Bmdsx* ^*F1*^ by the presence and absence of a novel exon, 3n and a known exon 5, respectively, and the detection of splice form *Bmdsx* ^*M3*^ confirms our earlier report of the existence of novel exon, 2n [31]. In addition, bands for some isoforms in Figure 1B are overlapped, and the relative abundance of previously reported forms are high in each lane for tissues of both sexes. The new isoforms are evidently at a lower abundance than that for the major previously described isoforms (Figure 1C and 1D), indicating that some new isoforms might be not functionally significant events. If the amount of an isoform is very low, it may be the by-product of splicing leakage, which can be tolerated due to its low frequency. We failed to find a clear band in Figure 1B for *Bmdsx* ^*F4*^, suggesting that *Bmdsx* ^*F4*^ may be not a functional event, and it may be a splicing error. Some female isoforms are also found in some tissues of males, which are consistent with the results described [30,31].

**Figure 3:**
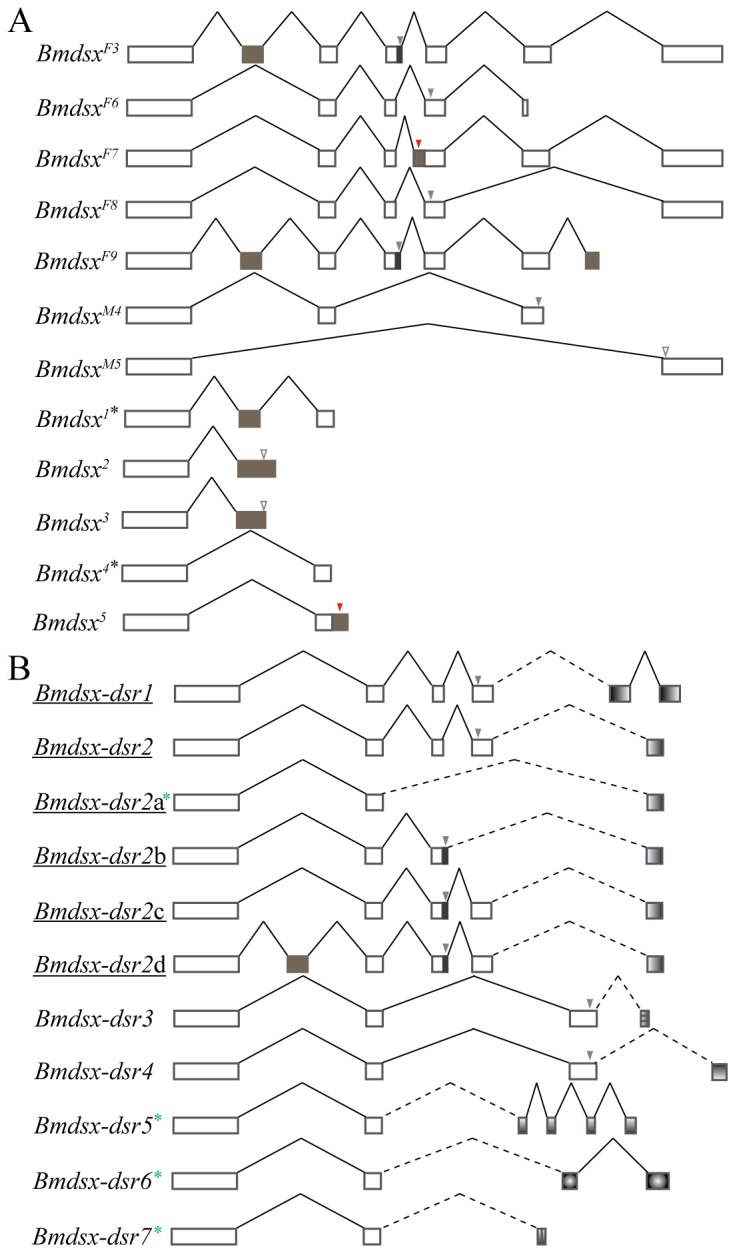
Novel alternatively-spliced and trans-spliced forms of *Bmdsx*. (A) Novel alternatively-spliced forms of *Bmdsx*. Boxes are *Bmdsx* exons, and the “V” lines indicate introns. Black and brown boxes are a stretch of 15 bp in exon 3 and novel exons, respectively. Grey and red triangles show the position of previously reported and newly-discovered stop codons, respectively. Hollow triangle shows the position of the newly-discovered stop codons in novel splice forms expected to be degraded by NMD. Black asterisk indicates the nonsense splice forms expected to be degraded by nonstop-mediated decay (NSD). (B) Novel putative trans-spliced variants of *Bmdsx*. Underlined splice forms indicate putative trans-spliced variants previously reported. Dashed “V” lines represent the potential trans-splicing events between *Bmdsx* transcripts and transcripts of other genes. The boxes downstream of the dashed “V” lines are the exons of *Bmdsx*-related genes, *Bmdsr*. Grey triangles indicate the position of reported stop codons. Green asterisks indicate the putative trans-spliced events, which occurs upstream of the termination codons.

Further verification of the presence of multiple splice forms of *Bmdsx* transcripts was obtained by performing 3' RACE using primers P3 and P4 that are specific to the first common exon, exon 1 (Figure 2B). A total of twelve novel, alternatively-spliced and five novel, putatively trans-spliced forms were recovered, cloned and analyzed (Figure 3). The identification of splice forms *Bmdsx* ^*F3*^ and *Bmdsx* ^*F9*^ provided secondary confirmation for the existence and use of exon 2n [31]. There were three splice forms found in males and females that included exon 2n, *Bmdsx* ^1^, *Bmdsx* ^*2*^ *and Bmdsx* ^*3*^ (Figure 3A). Splice forms *Bmdsx* ^2^ and *Bmdsx* ^3^ have 89-bp and 31-bp stretches at the 3' end of exon 2n followed by a polyA tail, respectively. *Bmdsx* ^4^ and *Bmdsx* ^5^ are also found in both males and females. *Bmdsx* ^4^ is also a nonsense splice form that includes only exon 2 while *Bmdsx* ^5^ includes exon 2 to which 138 bp were added at the 3' end. There were two splice forms in which exon 5, an exon common to all previously described transcripts in males and females, had been skipped, *Bmdsx* ^*F8*^ and *Bmdsx* ^*M5*^ which is also missing exons 2, 3 and 4. Splice form *Bmdsx* ^*F9*^ was identical to *Bmdsx* ^*F3*^ except exon 6 was replaced with novel exon 6n in *Bmdsx* ^*F9*^. *Bmdsx* ^*F6*^ was also missing exon 6 and was polyadenylated at the 5' end of exon 5, a situation also found in *Bmdsx* ^*M4*^. *Bmdsx* ^*F7*^ is identical to *Bmdsx* ^*F1*^ except that splicing of exon 4 occurred 133-bp upstream of the usual exon 4 splice acceptor site. These extra nucleotides contained a potential binding site of *Sex-lethal* (*Sxl*), TTATTTTTTATTTTCTTTTTT. In *Drosophila*, *Sxl* functions as the master regulatory gene regulating the alternative splicing of transformer (tra), which in turn promotes the splicing of exon 4 of *Dmdsx* transcripts in females [1,9,14]. It was reported that three negative splicing regulators BmPSI, BmHrp28 and BmIMP specifically bound to the CE1 element at the 5' end of exon 4 [37-39]. However, these three inhibitors might not be the master regulator upstream of *Bmdsx* in the sex-determining cascade in *B. mori*. The *B. mori* homolog of *DmSxl*, *BmSxl*, was identified previously [17] and while *BmSxl* may not serve the master regulator in *B. mori* as in other non-Drosophilid species. We cannot eliminate the possibility that *BmSxl* functions as a co-regulator of *Bmdsx*.

*Trans*-splicing is another method by which primary transcripts are processed in *B. mori* and *D. melanogaster*. *Trans*-splicing is involved in transcript processing of *mod* (*mdg4*) in *B. mori* and *mod* (*mdg4*) and *lola* in *D. melanogaster* [40,41]. However, it is important to be aware of the possibility that some putative trans-splicing products may be experimental artifacts. Although eleven putative trans-spliced variants of *Bmdsx* have been cloned by 3' RACE, including six trans-spliced variants reported previously (Figure 3B) [31], it is not clear at the moment whether these splice forms are the result of natural splicing events or are possible PCR artifacts arising from strand-switching.

### Novel exons and revised genomic organization of *Bmdsx*

**Figure 4:**
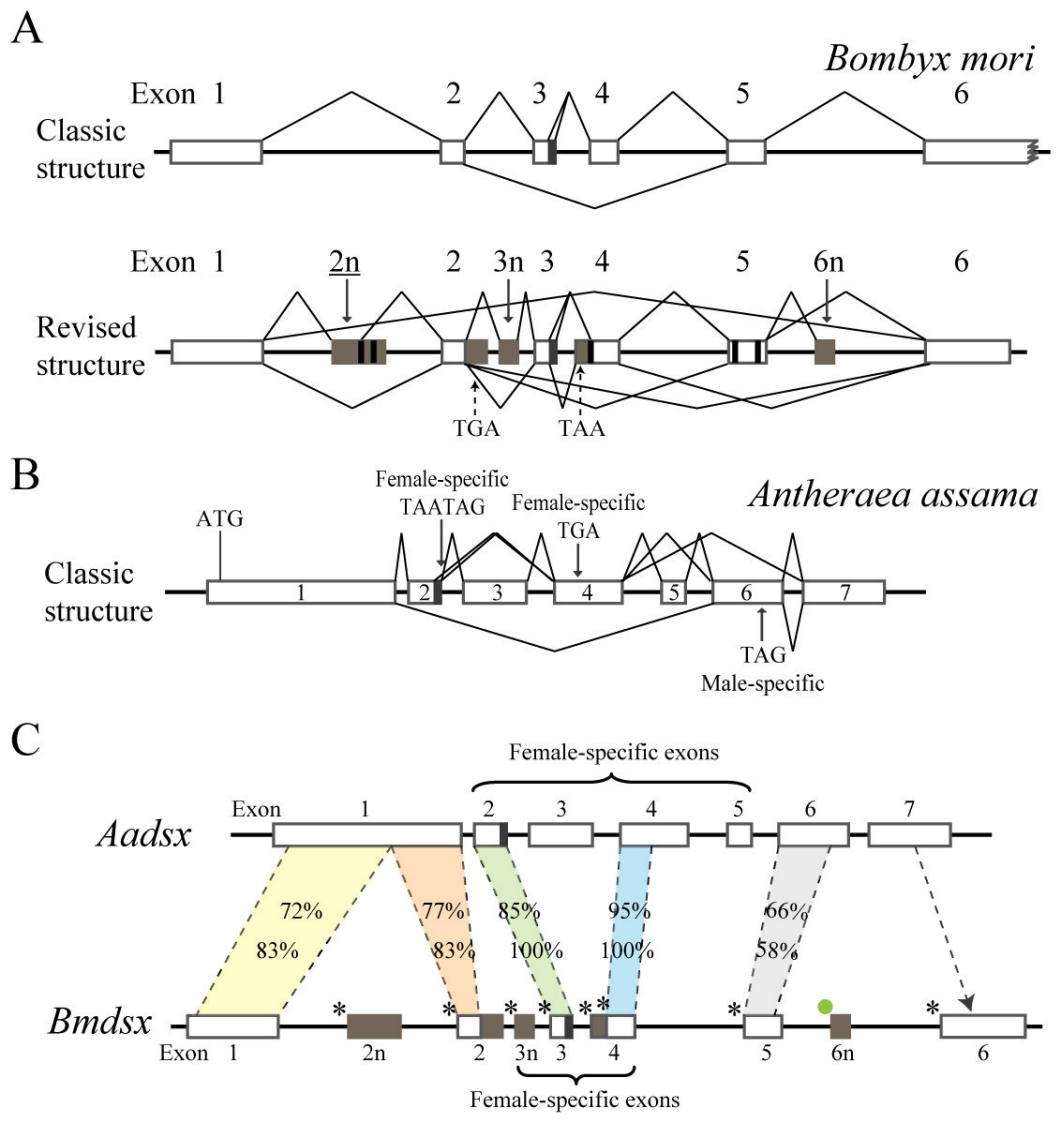
Classic and revised gene models and expression patterns of *Bmdsx*. (A) Classic and second revised gene model of *Bmdsx*. Boxes represent *Bmdsx* exons. Black boxes strand for astretch of 15 bp in exon 3, and brown boxes represent novel exons or extended regions of exons. 6n indicates a novel exon upstream of exon 6. Dashed vertical arrows show the position of stop codons. “V” lines indicate splicing events between two exons. The vertical lines within exons indicate the boundaries between different splice junctions leading to different splice forms. (B) Classic gene model of the *dsx* gene of *A. assama*, *Aadsx*. The black box shows the position of a 15-bp stretch of nucleotides in exon 2. Vertical arrows indicate the position of stop codons. (C) Comparative genomic structure of the *A. assama* and *B. mori* *dsx* genes. Percentages above and below represent the identities of the corresponding nucleic and amino acid sequences between *Aadsx* and *Bmdsx*, respectively. Asterisks and dot indicate the canonical and weak splicing acceptor sites, respectively. Brackets indicate the female-specific exons.

Previous analyses of *Bmdsx* showed that it is composed of six exons and five introns, including an alternative 5' splice site in intron 5 resulting in an additional 15-bp at the 3' end of exon 3. In our current understanding of the genomic organizationof *Bmdsx*, the 3' end of exon 6 is undetermined [19,30]. This classic understanding of the structure of *Bmdsx* can now be substantially revised in three ways in light of our findings (Figure 4A). First, the presence of novel exons 2n, 3n, and 6n has been confirmed by the successful identification of new splice forms containing these novel exons. Second, there is a 138-bp stretch at the 3' end of exon 2 and an alternative splice site that add 133 bp to the 5' end of exon 4. Third, exon 6 has been confirmed to be 525bp. Based on these observations we now know that *Bmdsx* consists of nine exons with eight introns that vary markedly in length from 287 to 55569 bp (Table 2). All of the splice sites in *Bmdsx* abided by the GT-AG rule [42] and an analysis of the 5' splicing sites of *Bmdsx* introns revealed that only those associated with introns 1 and 6 conformed to the consensus sequence GTRAGY [43]. In addition to minor deviations at the 5' splice sites of introns 4, 5 and 7, major deviations from the consensus GTRAGY were observed at the 5' splice sites of introns 2 and 3. Analysis of the 3' splice sites revealed that the length of the pyrimidine stretches associated with the 3' splice sites did not deviate significantly from the consensus number of 8.49±1.57 [19], and most of the 3' splice sites were canonical (Figure 4C, Table 2). However, the pyrimidine stretch preceding novel exon 6n deviated markedly from the consensus number (6/12), indicating that the 3' end of intron 7 contained a weak splice site, and its splicing might require a positive splicing regulator binding to exon 6n (Figure 4C, Table 2).

**Table 2:** Exon/intron boundaries of the *Bmdsx* gene.

Exon	Size (bp)	5' splice site	Intron	Size (bp)	3' splice site	N° of Y
1	67(5'UTR)+502	CC/**GT** AAGT	1	31300	TTTTTATGATTG A T**AG**/A	8
2n^a^	81	AG/**GT** ATTA	2	45831	TAATTTATTGTT A C**AG**/C	8
	112					
	170					
2^a^	144	AG/**GT** ACCG	3	2761	TAATCTGGTATT T C**AG**/G	7
	136					
	282					
3n	108	AG/**GT** AAGA	4	287	AGAGTCTTGTCC G C**AG**/G	7
3^a^	82	CA/**GT** ACGG	5^b^	4077	GATTTCCACTTT A C**AG**/T	9
				4204	ATTTTCTTTTTT G T**AG**/G	11
	97	TA/**GT** AATA		4189		
4^a^	169	GT/**GT** AAGT	6	55569	GTCCGGACCGTT A C**AG**/G	7
	296					
5^a^	218	AG/**GT** AAAC	7^c^	27810	TTATGCTGAAAC A A**AG**/G	6
	199					
	17					
6n	112		8	27479	TTTTTTTTAATT T C**AG**/A	10
6	525					
consensus					YYYYYYYYYYYY NY**AG**/G	8.49±1.57^d^

/ Diagonal line indicates the boundary between exon and intron. Left sequence, exon; Right sequence, intron.

^a^ Exons alternatively-spliced at different 5' splice sites.

^b^ Intron alternatively-spliced at different 3' splice or 5' splice sites.

^c^ Intron containing weak 3' splice site.

^d^ Average number of pyrimidines from 5 to 17 nucleotides upstream of 3' splice site in *B. mori* [19].

The revised structure of *Bmdsx* and its comparison to *Aadsx*, the *dsx* gene from *Antherea assama*a basal member of the superfamily Bombycoidea, suggests that the ancestral *dsx* gene gained and lost exons as well as gaining an intron. The *Aadsx* gene contains seven exons, including a 15-bp stretch at the 3' end of exon 2, and undergoes alternative splicing to produce seven transcripts (Figure 4B) [29]. The first exon of *Aadsx* is homologous to *Bmdsx* exons 1 and 2, suggesting that the ancestral form of *dsx* had an *A. assama*-type first exon that acquired an intron and exon 2n in the lineage leading to *B. mori*. The corresponding homologous relationship of the second, fourth and sixth exons of *Aadsx* to *Bmdsx* exons 3, 4 and 5 indicated that *Aadsx* third and fifth exons were lost, whereas the novel exons 3n and 6n were acquired in *B. mori* lineage. 

### Tissue and sex-specific expression

**Figure 5:**
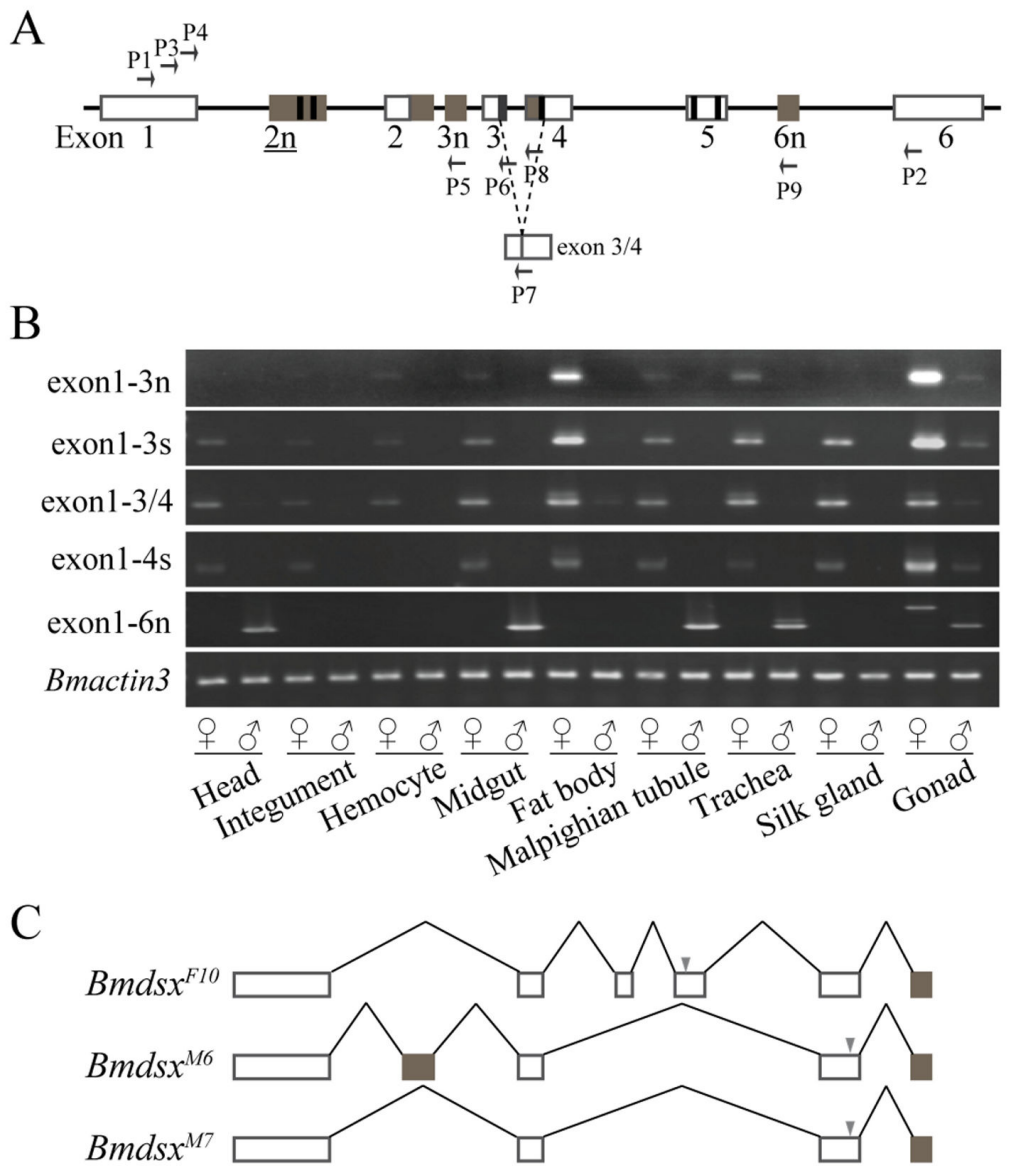
Expression and alternative splicing analysis of novel and sex-specific exons of *Bmdsx*. (A) Horizontal arrows indicate the position of specific primers on the revised gene model of *Bmdsx*. Dashed “V” lines indicate the splicing between exons 3 and 4 producing exon 3/4 at positions not including the 15 bp and 133 bp at the 3' and 5' ends of exon 3 and 4, respectively. (B) Analysis the sex and tissue specific patterns of expression of transcripts containing exon 1 and exons 3n, 3s, 3/4, 4s, 6n. 3n and 6n are novel exons upstream of exons 3 and 6, respectively. 3s and 4s are exons 3 and 4 with a stretch of 15 bp and 133 bp, respectively. (C) Novel alternatively-spliced forms of *Bmdsx* containing exons 1 and 6n and expressed in female gonads. Grey triangle indicates the position of stop codons.

Exon-specific primers were used to test for the presence of transcripts that included specific exons in the head, integument, hemocytes, midgut, fat body, Malpighian tubules, trachea, silk glands and gonads of both sexes (Figure 5A). Exon 2n was known to be expressed in both males and females [31] and it was not included in this analysis. Exon 3n was alternatively-spliced in a female-specific manner and transcripts containing this exon were readily detected. A low level of transcripts containing exon 3n was also detected in male gonads (Figure 5B). An identical pattern of alternative splicing and expression were seen for transcripts containing exons 3 and 4 as described below (Figure 5B), suggesting that the sex-specific splicing of exon 3n is controlled by an unknown regulatory factor upstream to *Bmdsx* in the sex-determination cascade. In *D. melanogaster*, exon 4 of *Dmdsx* contains a TRA/TRA2 binding site (dsxRE/PRE); no such sequences have been detected in exon 3n of *Bmdsx*. Because exon 3n contains a canonical 5' splice site, it suggests that a putative negative regulator was recruited to control splicing of exon 3n similar to exons 3 and 4. Exon 3 with the 15 bp 3' stretch (exon 3s), exon 4 with the 133 bp 5' stretch (exon 4s) and exon 3/4 arising from the splicing of exons 3 and 4 were all expressed in the female soma and gonads, as well as at low levels in the male gonads (Figure 5B) [19,30]. It is interesting to speculate that the gain or loss of the 133-bp stretch at the 5' end of exon 4 might be related to the recruitment of a Sxl-like factor since a Sxl-like binding site was found here. Although *Sxl* might not serve a master regulator in *B. mori* [10,11,17], considering the presence of a poly(U) tract, the RNA binding and splicing regulation of *tra* by Sxl protein and the autoregulation of *Sxl* itself [44], we suspect that the choice of the 3' splice site of intron 5 also involves a Sxl-like factor.

Exon 6n, as described earlier, is part of the 3' UTR of *Bmdsx* ^*F9*^ (Figure 3A). Our expression analysis of transcripts containing exon 6n showed that they were found almost exclusively in male tissue (Figure 5B). Exon 6n-containing transcripts were also detected in the gonads of females but the size of the 6n-containing transcripts were significantly larger than those detected in males (Figure 5B). Exon 6n-containing transcripts were isolated, cloned and sequenced, resulting in the discovery of three novel splice forms including one female (*Bmdsx* ^*F10*^) and two male (*Bmdsx* ^*M6*^ and *Bmdsx* ^*M7*^) splice forms (Figure 5C). *Bmdsx* ^*F9*^ was not detected under those conditions and we suspect that the sensitivity of our RT-PCR was such that if this transcript was present in only a few cells at a low level, we would have failed to detect it. In Drosophila, alternative splicing of *msl-2* 5'UTR is affected by the master regulator Sxl. Although alternative splicing does not affect the *msl-2* ORF, it represses the translation of the mRNA leading to the absence of MSL-2 in males [45]. The protein coding capacity of transcripts containing exon 6n is not changed and consequently we speculate that its use may be not only regulated by some key upstream gene in the sex-determining cascade, but also its presence likely modulates subsequent downstream information transfer in the sex-determination cascade.

Therefore, in summary, with the exception of *Bmdsx* ^*F10*^, all *Bmdsx* ^*F*^ transcripts are primarily female-specific in the soma and gonads, with low levels of expression in the male gonads, which does not affect testis development. *Bmdsx* ^*F10*^ is specifically expressed in the female gonads. Additionally, *Bmdsx* ^*M6*^ is specifically expressed in male trachea, while the remaining six *Bmdsx* ^*M*^s are specifically expressed in males. These expression patterns may be related to the sex-specific and tissue-specific functions of these different transcripts.

### BmDsx protein

When compared to *dsx* reported in other species, the situation in *B. mori* is complicated. Our observations beg the question as to the function of these various alternatively-spliced transcripts. To begin to address that question we have considered the potential of the transcripts isolated and analyzed here to produce proteins that might function in sexual development. Generally, a Dsx protein with regulatory function on downstream targets must contain several characteristics such as a common N-terminal region in both sexes, a sex-specific C-terminal region, a conserved DNA binding domain (OD1 domain) containing two overlapping binding sites with conserved cysteine and histidine residues, a conserved oligomerization domain (OD2 domain), the N-terminal region of the OD2 domain common to both sexes and containing some important nonpolar amino acids, the C-terminal region of the OD2 domain located in the female-specific C-terminal region, and a glycine located in the first position of the female-specific C-terminal region.

**Figure 6:**
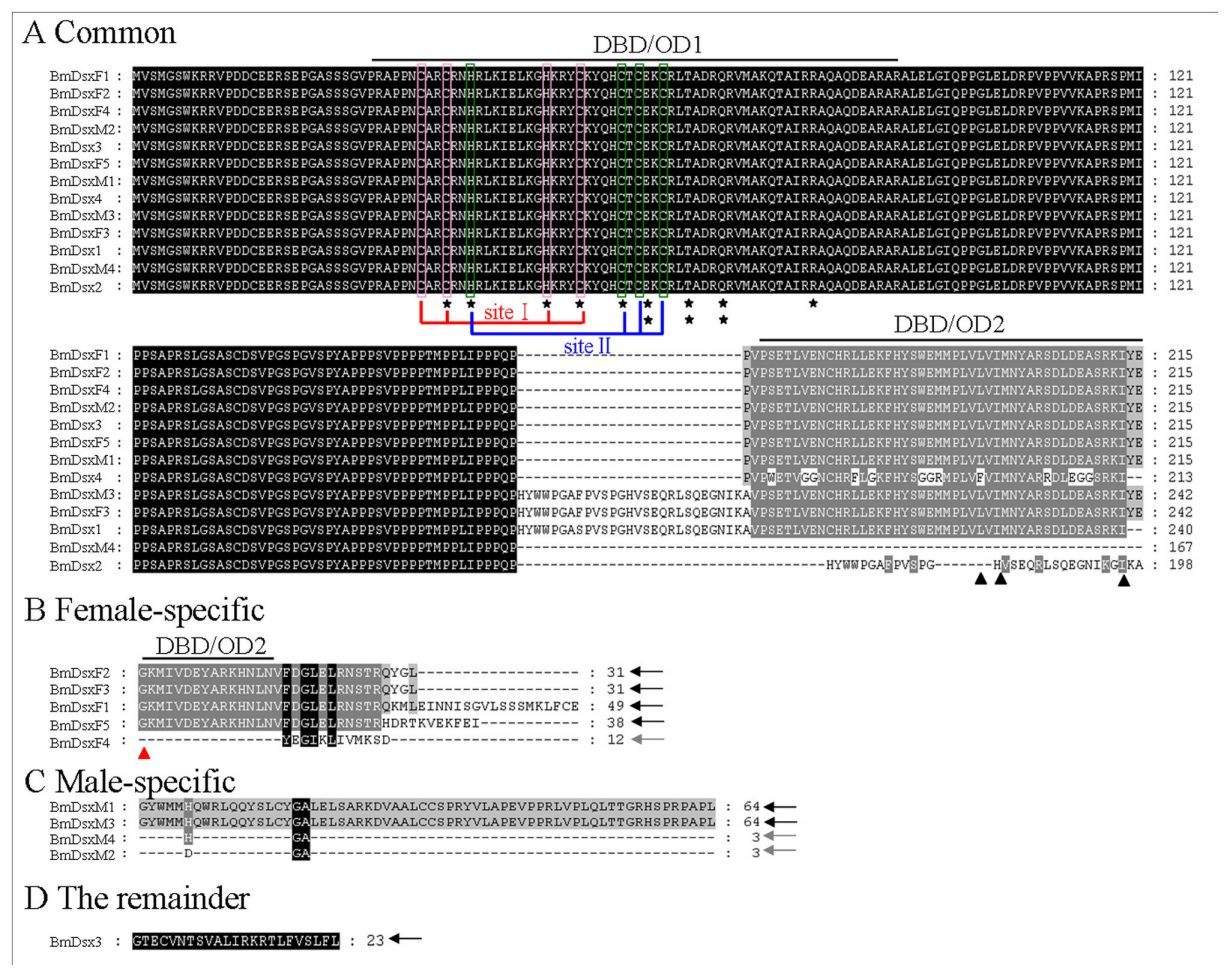
An alignment using ClustalX of the putative BmDsx proteins encoded by all the alternatively-spliced forms of *Bmdsx*. The sequence is divided into (A) the region common to both sexes, (B) the female-specific region, (C) the male-specific region and (D) the C terminal region common to females and males. Overline indicates the positions of the DNA-binding domain (OD1 domain) and the oligomerization domain (OD2 domain). Asterisks and triangles indicate the position of key residues in the OD1 and OD2 domains, respectively. The red triangle indicates the position of a conserved glycine, the first amino acid of female-specific C-terminal region. SiteⅠ(Red) and siteⅡ(Blue) indicate the positon of the two overlapping binding sites aligned with cysteines and histidines that coordinate Zn^2+^. Black arrow indicate the candidate Dsx proteins that have regulatory function on downstream targets, and grey arrow shows the Dsx proteins without regulatory function.

Of the ten female-specific *Bmdsx* transcripts, *Bmdsx* ^*F4*^ is predicted to result in a truncated nonfunctional protein, BmD*sx*F4, because of a premature termination codon (PTC) present in exon 3n (Figure 2A and 6, Table 3). Because PTC-containing mRNA can be targeted for degradation by nonsense-mediated mRNA decay (NMD) to avoid producing truncated proteins with potentially deleterious functions [46], we suspect that *Bmdsx* ^*F4*^ will be degraded by NMD during the first few rounds of translation. The acquisition of a PTC by exon 3n in females may be a method of regulating BmDsxF translation. The remaining nine female-specific *Bmdsx* transcripts are expected to result in two functional (BmDsxF1 and BmDsxF3) [31,32] and two potentially functional (BmDsxF2 and BmDsxF5) Dsx proteins, which have three different C-termini (Figure 6, Table 3). The C-terminus of Dsx protein generally serves a regulatory domain, and therefore different C-termini possibly cause BmDsxF proteins to have different effects on the control of their downstream target genes involved in female sexual development. BmDsxF1 is encoded by four transcripts, which use a common 5' end but differ at their 3' ends by the use of alternative polyadenylation sites. *Bmdsx* ^*F7*^ contains the 133-bp stretch at the 5' end of exon 4 which contains a termination codon resulting in the production of a novel female-specific C-terminus of 38 aa (Figure 3A and 6), which possibly causes BmDsxF5 to have a different effect on the control of downstream target genes related to female sexual development.

**Table 3:** Putative Dsx proteins and their corresponding splice forms.

Candidate protein	Splice form
BmDsxF1^a^	*Bmdsx* ^*F1*^, *Bmdsx* ^*F6*^, *Bmdsx* ^*F8*^,*Bmdsx* ^*F10*^
BmDsxF2^b^	*Bmdsx* ^*F2*^,*Bmdsx* ^*F5*^
BmDsxF3^a^	*Bmdsx* ^*F3*^, *Bmdsx* ^*F9*^
BmDsxF4	*Bmdsx^F4^*
BmDsxF5^b^	*Bmdsx^F7^*
BmDsxM1^c^	*Bmdsx* ^*M1*^, *Bmdsx* ^M4 f^, *Bmdsx* ^*M7*^
BmDsxM2	*Bmdsx^M2^*
BmDsxM3^d^	*Bmdsx* ^*M3*^, *Bmdsx* ^*M6*^
BmDsxM4	*Bmdsx^M5^*
BmDsx1	*Bmdsx* ^1 f^
BmDsx2	*Bmdsx* ^2 f^, *Bmdsx* ^3 f^
BmDsx3^e^	*Bmdsx5*
BmDsx4	*Bmdsx* ^4 f^

^a^ Female Dsx protein having regulatory function on downstream targets as described [31,32].

^b^ Female Dsx protein having potentially regulatory function on downstream targets.

^c^ Male Dsx protein having regulatory function on downstream targets as described [47].

^d^ Male Dsx protein having potentially regulatory function on downstream targets.

^e^ Common Dsx protein having potentially regulatory function on downstream targets.

^f^ Transcripts possibly resulting from internal priming or some unknown error.

Of the seven male-specific *Bmdsx* transcripts, two, *Bmdsx* ^*M2*^ and *Bmdsx* ^*M5*^, might result in truncated non-functional proteins. In the transcript of *Bmdsx* ^*M2*^, exon 5 is skipped (Figure 2A), resulting in a putative protein (BmDsxM2) without the male-specific C-terminus (Figure 6, Table 3). Translation of *Bmdsx* ^*M5*^ could result in a protein (BmDsxM4) without the male-specific C-terminus as well as the OD2 domain (Figure 6, Table 3). Consequently, we speculate based on the structure of these transcripts that *Bmdsx* ^*M2*^and *Bmdsx* ^*M5*^ will undergo nonsense-mediated degradation, avoiding the production of truncated proteins. Transcripts *Bmdsx* ^*M1*^, *Bmdsx* ^*M4*^ and *Bmdsx* ^*M7*^ only differ at their 3' ends (Figure 2A, 3A and 5C). We suspecte that *Bmdsx* ^*M4*^ will be degraded shortly after splicing because of the very short distance between the stop codon (TAG) and the poly(A) tail as well as a lack of a polyadenylation signal (AAUAAA). The origin of transcript *Bmdsx* ^*M4*^ is not clear though it appears not to have arisen from an internal priming event, due to the absence of a short-A stretch following the polyadenylation site, which eliminates nonspecific hybridization of oligo-d(T) to the short-A stretch as playing a role in the genesis of this transcript. The remaining male-specific splice forms are predicted to result in functional (BmDsxM1) [47] and potentially functional (BmDsxM3) Dsx proteins (Figure 6, Table 3). BmDsxM3 is different from BmDsxM1 by a 27-aa insertion between OD1 and OD2 domains, which may affect the binding affinity of BmDsxMs to downstream target sites.

Five splice forms were cloned from males and females, however, only one, *Bmdsx* ^5^, results in a potentially functional protein (BmDsx3). Despite the presence of a short-A stretch following the polyadenylation site, a consensus AAUAAA at 22 nucleotides upstream of the poly(A) tail indicates that *Bmdsx* ^5^ probably results from the genuine use of a polyadenylation site. Its validity needs to be tested further. Two of the remaining splice forms, *Bmdsx* ^1^ and *Bmdsx* ^4^, are expected to be degraded by nonstop-mediated mRNA decay (NSD) due to the absence of a translation termination codon [48]. These transcripts may have arisen as a result of internal priming because of the presence of a short-A stretch following the wrong polyadenylation site, providing a nonspecific annealing site for oligo-d(T). Putative protein BmDsx2 encoded by splice forms *Bmdsx* ^2^ and *Bmdsx* ^3^ lacks an OD2 domain and C-terminus (Figure 6, Table 3), and these splice forms may result from internal priming due to the presence of short-A stretches following incorrect polyadenylation sites. Consequently, we speculate that the protein BmDsx2 will not be synthesized.

 Therefore, in summary, besides the confirmed proteins BmDsxF1, BmDsxF3 and BmDsxM1 [31,32,47], four candidate Dsx proteins (Female-specific BmDsxF2 and BmDsxF5; Male-specific BmDsxM3; Male- and female-expressed BmDsx3) are expected from *Bmdsx* that have the potential to regulate the expression of downstream sexual development-related targets. In some cases, multiple splice forms differed only in their 3' UTR and there is the potential that these differences, while having no effect on the protein produced, could affect translation efficiency.

**Figure 7:**
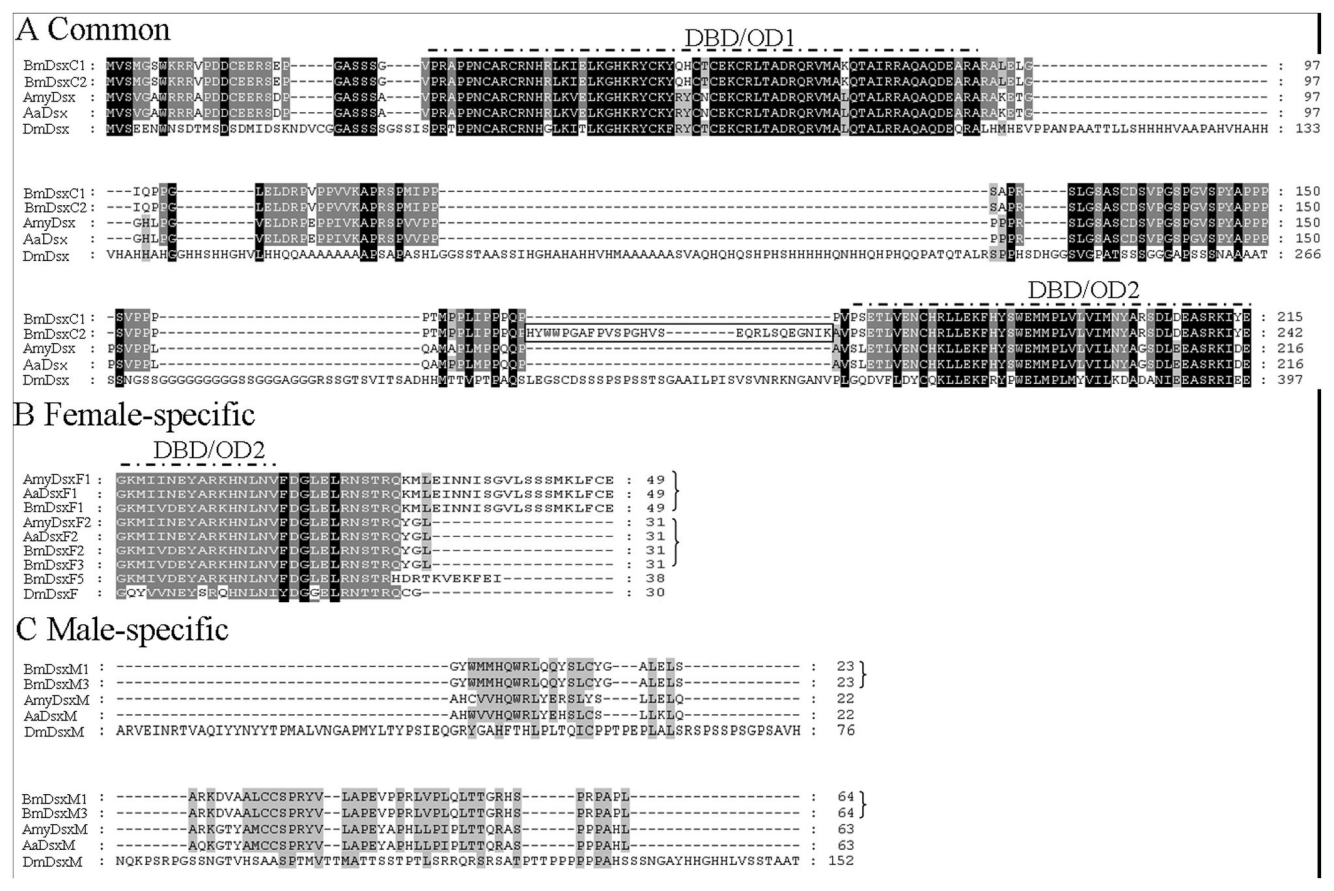
An alignment using ClustalX of Dsx proteins of *B. mori* (*Bm*), *A. mylitta* (Amy), *A. assama* (Aa) and *D. melanogaster* (*Dm*). Dashed line above the sequences indicates the positions of the conserved DNA-binding domain (OD1 domain) and oligomerization domain (OD2 domain). The rectangular box shows the position of 27 aa arising from exon 2n. The brackets indicate proteins with the same sex-specific C-termini.

Each of the six sex-specific BmDsx proteins (BmDsxF1, BmDsxF2, BmDsxF3, BmDsxF5, BmDsxM1, BmDsxM3) could be divided into three regions (Figure 6). Two were found in proteins from both males and females and the third was sex-specific. The amino terminus was common to all proteins and was either 215 or 242 amino acids in length containing the DNA binding domains OD1 and OD2. The sex-specific regions included the C-terminus which in females could be 31 aa, 38 aa or 49 aa in length while the male-specific C-terminal region was 64 aa. Comparison of the BmDsx proteins with the reportedly sex-specific Dsx proteins of *D. melanogaster*, *A. assama* and *A. mylitta* showed that OD1 and OD2 domains and the sex-specific C-termini were very conserved in the three Lepidopterans but some what diverged from *Drosophila* (Figure 7). The 27 aa arising from exon 2n was unique to *B. mori*. It was common for the three Lepidopterans to have the two female-specific C-termini of 31 aa and 49 aa while the female-specific C-terminus of 38 aa derived from the 133-bp stretch at the 5’ end of exon 4 was unique to *B. mori*.

In conclusion, the *dsx* gene in the domesticated silkworm displays characteristics that distinguish it from the other insects in which it has been studied. Seventeen different alternatively-spliced transcripts and eleven putative trans-spliced transcripts, potentially encoding for four female-specific, two male-specific and one shared functional Dsx protein. Understanding the roles and functions of these proteins in the sexual differentiation of *B. mori* is expected to enlighten our understanding of sexual differentiation in other organisms and the data reported here suggest that there is much more to be learned, reminiscent of *Sxl* and *dsx* in the model insect *D. melanogaster*.
